# Cytolethal distending toxin-producing *Escherichia coli* clinical isolates from Mexican children harbor different *cdt* types causing CDT-induced epithelial pathological phenotypes

**DOI:** 10.1007/s00430-025-00816-4

**Published:** 2025-02-02

**Authors:** Jazmin Huerta-Cantillo, Lucia Chavez-Dueñas, Mussaret Bano Zaidi, Teresa Estrada-García, Fernando Navarro-Garcia

**Affiliations:** 1 Department of Cell Biology, CINVESTAV-IPN, Av. IPN 2508, 07360 Mexico City, Mexico; 2 University Program in Epidemiological and Emerging Risks Research, UNAM, Mexico City, Mexico; 3 Department of Molecular Biomedicine, CINVESTAV-IPN, Av. IPN 2508, 07360 Mexico City, Mexico; 4 Infectious Diseases Research Unit, Hospital General O’Horan, Merida, Mexico

**Keywords:** *Escherichia coli*, Cytolethal distending toxin, *Cdt* types, Cell cycle arrest, Actin cytoskeletal remodeling, Nuclei enlargement, Cell distention, *E. coli* clinical isolates, *cdtABC* genes

## Abstract

Cytolethal distending toxins (CDTs), encoded by *cdtABC* genes, have DNase activity leading to cellular and nuclear distention, resulting in actin remodeling, irreversible cell cycle arrest and apoptosis of target cells. PCR *cdt*-positive *Escherichia coli* strains have been isolated from children with diarrhea worldwide. However, toxin production and biological activity of *cdt*^+^ strains are rarely confirmed. Here, we characterized the biological activity of *cdt*^+^ *E. coli* of clinical isolates from Mexican children with severe diarrhea and its relationship with the harbored *cdt* type. Ten isolates from seven patients containing *cdt*^+^ *E. coli*, one isolate from a patient containing *cdt*^*−*^* E. coli*, and a prototype CDT-producing *E. coli* were used to determine the harbored *cdt*-type, cell distention, actin remodeling and cell cycle arrest on epithelial cells. Three isolates harbored *cdt* type I, one type II, two type III, two type IV and two simultaneously type II and III. Lysates from eight *cdt*^+^ *E. coli* isolates caused cell distention, actin cytoskeletal remodeling and cell cycle arrest but two isolates from the same patient harboring simultaneously *cdt* type II/III did not. The *cdt* genes were necessary and enough to cause the cytolethal distending pathology. Mutants in *cdtAB*_*I*_*C* (O86:H34 strain; *cdt-*I) and *cdtAB*_*II*_*C* (isolate; *cdt*-II) were complemented by *cdtAB*_*II*_*C* genes and both recovered the CDT-induced phenotypes. Transformation of *E. coli* BL21 by *cdtAB*_*II*_*C* genes caused this cytolethal distending pathology. These data indicate that *cdt* + *E. coli* isolates are potentially dangerous bacteria to cause serious epithelial cell damage and cell death to aggravate childhood diarrhea.

**Supplementary Information:**

The online version contains supplementary material available at 10.1007/s00430-025-00816-4.

## Introduction

Cytolethal distending toxin (CDT) was first discovered by Johnson and Lior as a new type of toxin in *Escherichia coli* strains isolated from patient with diarrhea in 1987 [1]. Since then, the presence of CDTs has been reported in various Gram-negative bacteria, such as *Aggregatibacter actinomycetemcomitans*, *Campylobacter* spp., *Escherichia albertii*, *Haemophilus ducreyi*, *Helicobacter* spp., and *Shigella* spp. [2]. CDTs are heterotrimeric holotoxins that are produced from three linked genes, *cdtA*, *cdtB*, and *cdtC*, which encode proteins (CdtA, CdtB, and CdtC) with predicted molecular masses of around 25, 30, and 20 kDa, respectively [3]. CDT intoxication of epithelial cells leads to nuclear and cytoplasmic distention, formation of actin stress fibers and nuclear fragmentation, resulting in irreversible cell cycle arrest (in G2/M) due to the maintenance of mitosis promoting factor in an inactive form and apoptosis in the target cells [4]. Even though all CDTs have the same enzymatic activity, the CDT variants produced by different bacterial species might vary regarding receptor specificity and internalization pathway, but in general the CdtA and CdtC subunits function as a ligand for cellular receptor(s), while CdtB is the active subunit that induces DNA double-strand breaks [3, 4].

In addition, five types of CDT (CDT-I through -V) have been reported in *E. coli* based on the amino acid sequence variation and its gene location. The *E. coli* CDTs can be divided into two groups: one of CDT-I and CDT-IV and the other by CDT-II, CDT-III and CDT-V, the intra-group and inter-group identities are ∼89% and ∼50%, respectively [2]. EcCDT-I and EcCDT-II were originally identified in enteropathogenic *E. coli* (EPEC) strains isolated from patients with diarrhea [5, 6]. EcCDT-III was found in an *E. coli* strain isolated from a calf with septicemia [7]. EcCDT-IV was discovered in pathogenic *E. coli* strains isolated from human or animal intestinal and extraintestinal sources [4] and EcCDT-V was detected in Shiga toxin-producing *E. coli* (STEC) or enterohemorrhagic *E. coli* (EHEC) strains [8]. Despite this high CDT diversity and of several reports detecting *cdt* genes by PCR, there is scant reports on the activity of this toxin on epithelial cells.

Although several studies regarding the isolation and characterization of CDT-producing bacteria from patients with diarrhea have been reported [9–16], the role of CDT in human diseases, including diarrhea, has not yet been established. CDT-encoding genes have been identified in both diarrheagenic and uropathogenic *E. coli* strains, as well as in those that do not belong to any recognized pathotype [13, 17, 18]. However, there is scant information on toxin production and biological activity of *cdt*^+^ strains and are rarely confirmed, as well as the correlation with the prevalence and clinical presentation of diarrheal disease caused by these CDT-producing *E. coli* strains. Here we show that several isolates of *cdt*^+^ *E. coli* from seven children with diarrhea, which had been clinically diagnosed [19], were capable to produce CDT of the different *cdt* types. Lysates from *cdt*^+^ isolates were able to cause the CDT pathological effects on epithelial cells, except for two isolates from the same patient. The pathological CDT effects included cell distention, actin cytoskeleton remodeling, nuclei enlargement and cell cycle arrest in G2/M phase. These effects were lacking in a *cdtABC* mutant and recovered by complementation. A *cdt*-II from an isolate was able to complement also a *cdtABC* (*cdt*-I) mutant of a prototypical strain and this exchange of *cdt* types enabled full CDT effects.

## Materials and methods

### Bacterial cultures

Bacterial strains and *E. coli* isolates were incubated in 25 ml of Lysogenic Broth medium at 37 °C overnight with shaking. Bacterial cultures were centrifuged at 5000×*g* for 20 min at 4 °C. Bacterial pellets were resuspended in 3 ml of cold PBS and sonicated four-times for 35 s at a 70% amplitude. The sonicated samples were clarified at 6000×*g* for 25 min at 4 °C. These clarified samples were sterilized throughout 0.22-μm-pore-size cellulose acetate membrane filters (Corning) and protein concentration was quantified by using the micro-Bradford method. Lysates were aliquoted and stored at −20 °C until use.

### Cell cultures

HEp-2 cells (ATCC-CCL23) were cultured in Dulbecco’s Modified Eagle Medium (DMEM) containing 2 mM L-glutamine, 1.5 g/l sodium bicarbonate, 1 mM sodium pyruvate, 0.1 mM non-essential amino acids (GIBCO, USA), 10% fetal bovine serum (ByProductos, Mexico), 100 U/ml penicillin and 100 μg/ml streptomycin (PAA Laboratories, Austria), and cultures were kept in 25 cm^2^ bottles at 37 °C in 5% CO_2_.

### Cytotoxicity assays

For the cytotoxicity assays, 1.1 × 10^4^ HEp-2 cells were seeded in 48-well microplates and incubated at 37 °C for 24 h in 5% CO_2_. Then, the old media was removed and a fresh DMEM medium containing bacterial lysates at different concentrations (50, 75, 100, 150, 200, 250, 300 and 350 μg/ml) was added and incubated for 24 h. Lysates from *E. coli* O86:H34 prototypical strain were used as a positive control and lysates from an 11-85D clinical isolate (*cdt*^−^) as a negative control. The culture medium was changed every 24 h by fresh medium without bacterial lysates until reaching 72 h of incubation. After this time, the cells were washed twice with PBS, fixed with 4% paraformaldehyde for 20 min, washed again and stained with Giemsa for 1 h. Each well was analyzed under light microscopy, and the optimal titer of toxin was taken as the minimal concentration of bacterial lysates (μg/ml) required to cause cell distention of 70–80% of the cell population without causing cell death.

### CDT biological activity assays

For the biological activity assays, HEp-2 cells were seeded in 8-well Lab-Tek chamber slides (Nalgene Nunc, USA) at a density of 1.2 × 10^4^ cells/well using the same protocol as that of cytotoxicity assays and the optimal lysate titers previously stablished for each bacterial isolate. After Giemsa staining, the cell size was calculated in each experimental condition. For this purporse, 10 photos were randomly taken with a 3000 × magnification for measuring the length of 50 cells/photo. The length of 500 cells for each bacterial lysate was averaged and these values were plotted using GraphPad Prism version 5.01. In the plot, data are shown as the mean ± SD. Statistical comparison of data was performed with an ANOVA One-Way/Dunn’s multiple comparison test. Values of *p* < 0.001 were considered statistically significant.

### FAS (fluorescent actin staining)

For FAS assays, HEp-2 cells were seeded in 8-well Lab-Tek chambers (Nalgene Nunc, USA) at a density of 1.2 × 10^4^ cells/well using the same protocol as that of biological activity assays. Once fixed, the cells were processed for actin staining by washing 5 times with PBS and blocking for 30 min with 1% bovine serum albumin (BSA) (Research Organics, USA). The actin cytoskeleton (F-actin) was then stained with rhodamine phalloidin, and TO-PRO was used to visualize the cell nuclei (both from Invitrogen, USA), incubated 40 min at room temperature in the dark. Finally, the cells were washed 5 times with PBS, twice with distilled water, and left to dry at room temperature in the dark. The slides were mounted in VectaShield (Vector, USA) medium by covering them with cover slides. Samples were analyzed under a confocal microscopy TCS-SP8 (Leica Microsystems, Wetzlar, Germany) and representative photos of each condition were taken using a 63 × objective and a 1.2 digital zoom.

### Inhibition of cell cycle analyses

To record the arrest of the cell cycle induced by CDT, HEp-2 cells were seeded in 35-mm Petri dishes (Corning, USA) at a density of 2.2 × 10^5^ cells and incubated at 37 °C for 24 h in 5% CO_2_. The old media was removed and a fresh DMEM medium containing bacterial lysates at the optimum concentration for each isolate was added and incubated for 48 h (medium was changed each 24 h by fresh culture medium without lysates). After the incubation time, the cells were washed twice with PBS, detached using 1 ml of 0.05% trypsin–EDTA, and then diluted in 2 ml of cold PBS and centrifuged at 240×*g* for 15 min at 4 °C. The cells were fixed overnight at −20 °C in 70% ethanol. Ethanol was then removed by centrifugation at 400×*g* for 10 min at room temperature and cells were resuspended in 0.25 ml of 0.06 mg/ml RNase A (Sigma, St. Louis, MO) in PBS and incubated at 37 °C for 1 h. Cells were stained with 30 μg/ml propidium iodine at 37 °C for 1 h in the dark. Cells (2 × 10^4^) were analyzed by a FACSCalibur flux cytometer (Becton Dickinson). Cell cycle analysis was performed by a univariate analysis using FlowJo_V10 software (Tree Star Inc., USA), where 2N values represent the cell population percentage in phase G0/G1, S represents the percentage in phase S and 4N the percentage in phase G2/M. In the plot, the averages from four independent experiments ± standard deviation (SD) are shown. The data were plotted using GraphPad Prism version 5.01software and the statistical data comparison was performed using ANOVA One-Way/Bonferroni’s multiple comparison test. Values of *p* < 0.001 were considered statistically significant.

### Bacterial adherence assay to HEp-2 cells

To evaluate the adherence pattern to epithelial cells, HEp-2 cells were seeded in 48-well plates (JetBiofil, China) at a density of 4 × 10^4^ cells in DMEM and incubated at 37 °C for 24 h in 5% CO_2_. Before infection, the cells were washed three times with DMEM without supplements. Previously, bacteria were grown in 2 ml of LB overnight at 37 °C at 150 rpm. An OD_600nm_ of culture was recorded to calculate a MOI of 10 for control bacteria, and a MOI of 25 for isolates to infect the cells. The cells were infected with these different MOI and incubated at 37 °C for 4 h in 5% CO_2_. The infected cells were washed twice with DMEM without supplements and fixed with 4% PFA at room temperature for 20 min and then washed twice with PBS. The cells were stained with a 1:10 ratio of Giemsa:PBS for 2.5 h and photos were taken at 600x.

### Typification of the ***cdt***^+^***E. coli*** isolates

All bacterial isolates and the prototypical O86:H34 strain (used as a positive control) were typed according to the types *cdt*-I, *cdt*-II, *cdt*-III and *cdt*-IV by PCR [4]. Briefly, a bacterial colony in 20 µl sterile water was boiled for 10 min and then centrifuged 14,000 rpm for 5 min. For the PCR, 3 µl of bacterial supernatants were used as well as the following components: 0.2 mM DTPs, each one, (Invitrogen), 1X Taq DNA polymerase buffer, 0.4 µM forward and reverse oligonucleotides, 1.5 mM MgCl_2_ and 1 U Taq DNA polymerase (Invitrogen) at a final volume of 25 µl. The amplification reaction was as follows: an initial denaturation (94 °C for 5 min), 30 cycles of denaturation (94 °C for 1 min), annealing (55 °C for 1 min), extension (72 °C for 1 min), and with a final extension (72 °C for 10 min). The PCR products were detected in 1.5% agarose and stained with ethidium bromide.

Oligonucleotides were previously described [4]. For *cdt*-I type, the primers cdt-IF and cdt-IR were used and produce an amplicon of 411 bp (Table 1). For *cdt*-II type, the primers cdt-IIF and cdt-IIR were used and produce an amplicon of 556 bp. For *cdt*-III the primers cdt-IIIF and cdt-IIIR were used and produce an amplicon of 555 bp. For *cdt*-IV the primers cdt-IVF and cdt-IVR were used and produce an amplicon of 350 bp (Table 1).

**Table 1:** Characteristics of primers used in this study

Name	Sequence	Prod^a^	References
cdt-IF	5′-CAA TAG TCG CCC ACA GGA-3′	411	Toth et. al. [4]^b^
cdt-IR	5′-ATA ATC AAG AAC ACC ACC AC-3′		Toth et. al. [4]^b^
cdt-IIF	5′-GAA AGT AAA TGG AAT ATA AAT GTC CG-3′	556	Toth et. al. [4]^b^
cdt-IIR	5′-TTT GTG TTG CCG CCG CTG GTG AAA-3′		Toth et. al. [4]^b^
cdt-IIIF	5′-GAA AGT AAA TGG AAT ATA AAT GTC CG-3′	555	Toth et. al. [4]^b^
cdt-IIIR	5′-TTT GTG TCG GTG CAG CAG GGA AAA-3′		Toth et. al. [4]^b^
cdt-IVF	5′-CCT GAT GGT TCA GGA GGC TGG TTC-3′	350	Toth et. al. [4]^b^
cdt-IVR	5′-TTG CTC CAG AAT CTA TAC CT-3′		Toth et. al. [4]^b^
cdt∆ABC-F	5′-GTG GAT AAA AAA CTA ATT GCA TTT TTG TGC ACA CTT ATT GTA GGC TGG AGC TGC TTC G-3′	1505	Delete *cdtABC*-I This study
cdt∆ABC-R	5′-TCA GCT CGT TAA TGG AGA CAT TAT TGC CGG AGA TAA TGG TGC ATA TGA ATA TCC TCC TTA G-3′		Delete *cdtABC*-I This study
cdt-II-FRT-F	5′-GGC CAA ATA CGA TAC AGT TTC GCA ATG TAG ACG TTG GTA CCT GTA TGA CAA TGT GTA GGC TGG AGC TGC TT-3′	1522	Delete *cdtABC*-II This study
cdt-II-FRT-R	5′-CTA AGG AGG ATA TTC ATA TGG ATC AAA GCG GGT GGA TAA TGA TTC GAA CGC CAA ACA CAG ACC A-3′		Delete *cdtABC*-II This study
cdtABCII-BamHI-F	5′-GCG CGG ATC CAA TGG CTA AYA AAY GYA CAC CTA-3′	2153	Cloning This study
cdtABCII- PstI-R	5′-TGG TTC TGC AGA ATA ATA GGC GAT TCA GTA TTT AAT GGG-3′		Cloning This study
k2	5′-CGG TGC CCT GAA TGA ACT GC-3′	470	Datsenko and Wanner [20]
Kt	5′-CGG CCA CAG TCG ATG AAT CC-3′		Datsenko and Wanner [20]
pTrcHis-F	5′-GAG GTA TAT ATT AAT GTA TCG-3′	2367	Cloning verification
pTrcHis-R	5′-GAT TTA ATC TGT ATC AGG-3		Cloning verification

^a^base pair

^b^*cdt* typing

### Deletion of *cdtABC* in *E. coli* E6468, O86:H34, (*cdt*-I) and the 08-184 isolate (*cdt*-II)

Deletion of *cdtABC* genes was performed using the technique for inactivating genes using PCR products [20]. *cdt* genes were replaced by a kanamycin resistance gene, which was generated by PCR. This resistance gene was synthetized using homologous sequences to the internal end regions of the genes being deleted (Table 1). The pKD4 was used as a template for the kanamycin resistance gene. The PCR conditions were as follows: 5 cycles of denaturalization (94 °C for 1 min), annealing (50 °C for 1 min), and extension (72 °C for 90 min), then 30 cycles of denaturation (94 °C for 1 min), annealing (65 °C for 1 min), and extension (72 °C for 2 min and 30 s), with a final extension (72 °C for 5 min).

The oligonucleotides used were cdt∆ABC-F and cdt∆ABC-R to amplify the kanamycin resistance gene for the prototypical *E. coli* E6468 (O86:H34) strain and cdt II-FRT-F and cdt II-FRT-R for the 08-184 lysate (Table 1). The first set of primers was designed using the known sequence of the *cdtABC* genes (access U03293.1). For the unknown sequence of *cdt* isolate 08-184, 27 *cdt* homologous sequences (BLAST, NCBI) were analyzed using CLUSTAL W alignment software. The oligonucleotide candidates were analyzed using Oligo Analyzer 1.1.2 software (University of Kuopio, Finland) to choose those representing the best characteristics: cdtII-FRT-F and cdtII-FRT-R (Table 1).

Two PCRs were used to verify the lack of the *cdtB* gene. The first one was performed using the cdt-IF and cdt-IR for the O86:H34 strain (*cdt*-I), and cdt-IIF and cdt-IIR for the clinic isolate 08-184 (*cdt*-II) (Table 1). Amplification was performed with 30 cycles of denaturation (94 °C for 1 min), annealing (55 °C for 1 min), and a final extension (72 °C for 10 min). The PCR amplicons were analyzed in 1.5% agarose gel stained with ethidium bromide. The second was performed using k2 and kt primers for the kanamycin resistance gene (Table 1) [20]. Amplification was performed with one cycle of denaturation (94 °C for 2 min), 30 cycles of denaturation (94 °C for 1 min), annealing (63 °C for 45 s), extension (72 °C for 1 min and 30 s), and a final extension (71 °C for 10 min). The PCR amplicons were analyzed in 1.5% agarose gel stained with ethidium bromide.

### Cloning of ***cdtAB***_***II***_***C*** from 08-184 isolate

For cloning the *cdtABC* genes from the clinical isolate 08-184 in pTrcHis2B, 13 *cdtABC* type II sequences were obtained from NCBI BLAST, and analyzed with alignment software CLUSTAL W. Two oligonucleotides were obtained: a degenerate forward primer cdtCII-BamH1-F for introducing a *BamH*I site and a reverse primer cdtABC-PstlI-R for introducing a *Pst*I site. This primer set was used to amplify *cdtAB*_*II*_*C* genes from the clinical isolate 08–184 by using the following protocol: an initial denaturation (94 °C for 3 min), 30 cycles of denaturation 95 °C for 30 s), annealing (60 °C for 30 s) extension (72 °C for30 s), and a final extension (72 °C for 10 min). The PCR products were detected in 1% agarose stained with ethidium bromide.

The PCR products (2,153 bp) were purified from a 1% agarose gel using QIAquick gel Extraction Kit (Qiagen) and were digested by *BamH*I and *Pst*I. *cdtAB*_*II*_*C* genes were cloned by ligation in pTrcHis2B (Invitrogen). The construction was used to transform *E. coli* BL21[DE3]pLysS. This construction was verified by detecting *cdt* type II by PCR (as described above) as well as by detecting the released insert (2,153 bp) using *BamH*I and *Pst*I, and finally by sequencing the whole construction was done by Plasmidsaurus (Eugene, OR).

For complementation assays, *cdtAB*_*II*_*C* construction was introduced in a *cdtAB*_*II*_*C* mutant of isolate 08–184 or the *cdtAB*_*I*_*C* mutant of strain O86:H34 by electroporation. Each bacterial complementation was verified by amplifying *cdtAB*_*II*_*C* by PCR (as described above).

## Results

### Clinical ***cdt***^+^***E. coli*** isolates cause cell distention

**Fig. 1:**
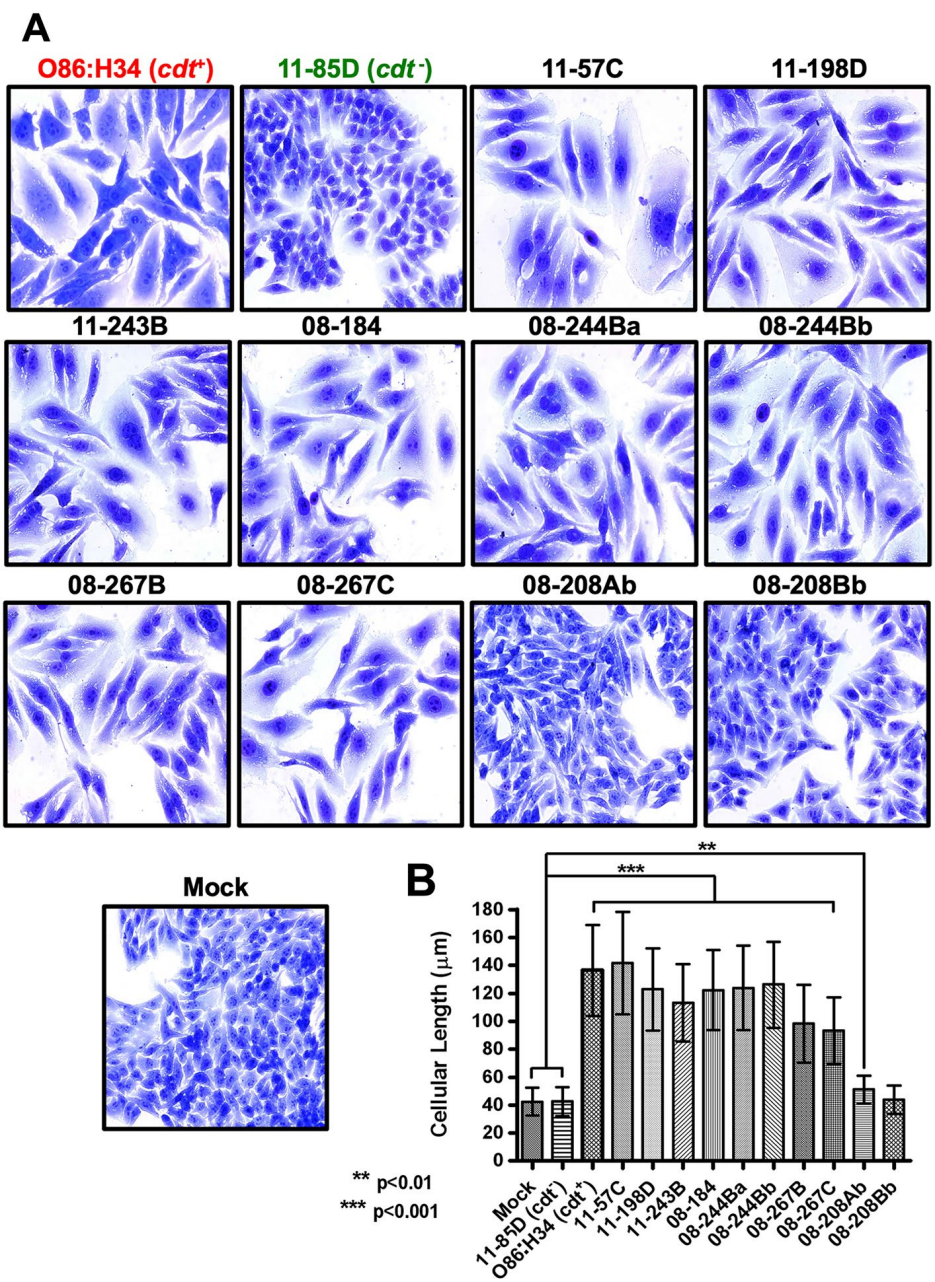
Lysates from clinical *E. coli* isolates cause cell distention. **A** Epithelial cells increase their cell size after incubation with filtered bacterial lysates. HEp-2 cells were incubated with filtered bacterial lysates at the appropriated MCC (see Table 2) for 24 h and then kept in fresh media. After 48 h post-interaction, cells were fixed with methanol and stained with Giemsa, then analyzed by light microscopy. Cells without bacterial lysates (Mock) and with lysates from a *cdt*^−^ strain (11-85D) were used as negative controls, and O86:H34 strain was used as a positive control. Most of the clinical isolates harboring *cdt* genes caused cell distention (11-57C, 11-198D, 11-243B, 08-184, 08-244Ba, 08244Bb, 08-267B and 08-267C), while two isolates (08-208Ab and 08-208Bb) did not cause cell distention (two isolates from the same patient). **B** quantification of cell distention induced by the lysates from *E. coli* isolates. Measure of the cell size in 500 cells from three independent experiments (statistically significant difference among the bacterial strains was performed by ANOVA One-way/Dunn’s multiple comparison test)

Seven children suffering diarrhea identified, in The Pediatric Emergency Department (PED) of the Hospital General O’Horan in Merida Mexico, during two different studies conducted from January 2007 to January 2009 and from January 2010 to July 2014 were selected to be exclusively positive for CDT-producing *E. coli* strains (*cdt*^+^) and negative for all other conventional pathogens (rotavirus, parasites or other bacteria) [19]. From these seven children (including Mayan and Mestizo boys and girls, between 3 and 38-months-old), ten *cdt*^+^ positive *E. coli* isolates were assessed for their capacity to cause cell distention on epithelial cells. An isolated identified as *cdt*^−^ was used as a negative control and the *E. coli* O86:H34 prototype strain was used as a positive control. Lysates from these bacteria were incubated for 24 h in Lab-Tek slide chambers containing a monolayer of HEp-2 cells, untreated cells were used as a negative control HEp-2 cells (Mock cells). The washed cells were analyzed by light microscopy 48 h post-incubation after fixing and staining them with methanol and Giemsa, respectively. All 10 *E. coli* isolates (11-57C, 11-198D, 11-243B, 08-184, 08-244Ba and 08-244Bb [both from the same patient], 08-267B and 08-267C [same patient] and 08-208Ab and 08-208Bb [same patient]), caused cell distention by increasing the cell size by around three times, except for 08-208Ab and 08-208Bb, both isolates were from the same patient (Fig. 1A). The quantitative analysis showed that the difference between the control cells (~ 40 μm) and those treated with the *cdt*^+^ lysates (~ 125 μm) was statistically significant (Fig. 1B); interestingly, one of two isolated (08-208Ab), previously classified as negative by the naked eye, was also statistically different but the size increase was marginal. These results were reproducible by using concentrated supernatants (~ 90 times) from the bacterial isolates (data not shown), suggesting that CDT is secreted to the bacterial extracellular medium.

**Table 2:** Minimum concentration of bacterial lysates to cause distension in 90% of the cell population

Bacterial lysates	(Mean ± SD) μg / μl
11-85D_(*cdt−*)_	NCD
O86:H34_(*cdt*+)_	0.100 ± 0.020
11-57C	0.050 ± 0.020
11-198D	0.150 ± 0.050
11-243B	0.150 ± 0.050
08-184	0.050 ± 0.025
08-244Ba	0.100 ± 0.020
08-244Bb	0.075 ± 0.015
08-267B	0.300 ± 0.050
08-267C	0.300 ± 0.050
08-208Ab	NCD*
08-208Bb	NCD

NCD does not cause cell distension. They were also used at 0.350 μg/μl

NCD* does not cause distension but slightly elongates cells compared to Mock p < 0.01

To determine the minimum concentration of *cdt*^+^ bacterial lysate required to cause cell distention, different concentrations of sterile bacterial lysates were used on the HEp-2 cell monolayer. Minimum concentrations of about 50 to 300 μg/ml were shown to cause cell distention. The lysates from clinical isolates 11-57C and 08-184 were more efficient (50 μg/ml) causing twice as much cell distention as the prototypical O86:H34 strain (100 μg/ml). The lysates from 08-244Ba and 08-244Bb isolates (from the same patient) also showed good potency to cause cell distention (75 and 100 μg/ml) followed by the 11-98D and 11-243B lysates (150 μg/ml). In contrast, lysates from 08-208Ab and 08-208Bb isolates were unable to cause cell distention even 350 μg/ml, with only slight cell elongation observed for the former.

### Clinical ***cdt***^+^***E. coli*** isolates harbor different types of ***cdtB***

**Fig. 2:**
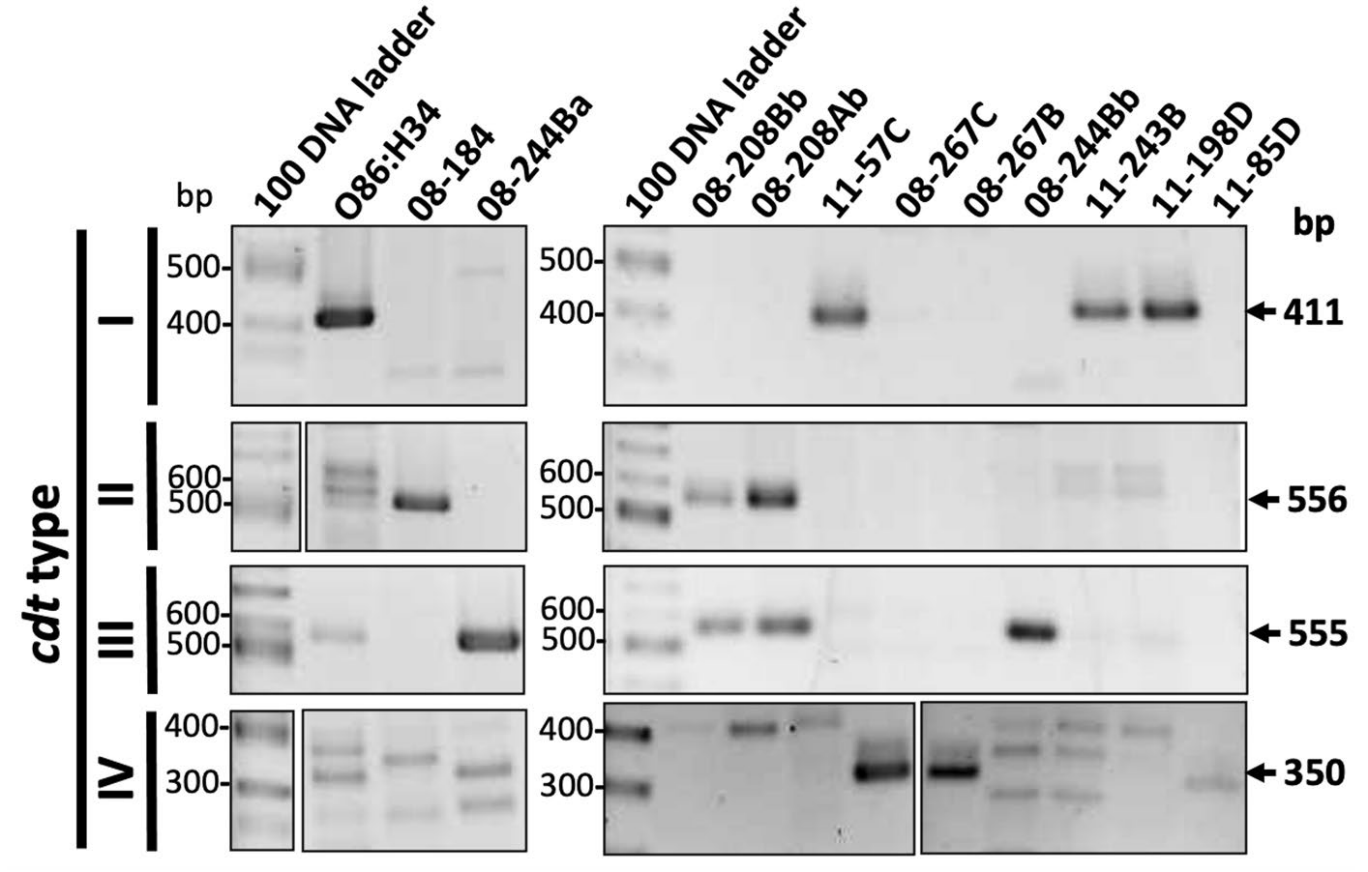
Variability of *cdt* type in the clinical *E. coli* isolates. Detection of the type of *cdt* genes by PCR using specific primers for *cdt* type I, II, III and IV (see Table 1)

To determine the type of *cdtB* harbored by the different *cdt*^+^ *E. coli* isolates a PCR was performed by using the previously reported primers [4] as shown in Table 1. Colony PCR analyses showed that 11-57C, 11-243B and 11-85D isolates produced a PCR amplicon of 411 bp indicating that they harbored *cdt* type I as the prototypical O86:H34 strain (Fig. 2). The 08-184 isolate produced only a PCR amplicon of 556 bp indicating that it harbored *cdt* type II, while the 08-267C and 08-267B isolates (from the same patient) produced a PCR amplicon of 350 bp indicating that they harbored *cdt* type IV. Interestingly, in the case of the *cdt* type III, we found two isolates from the same patient (08-208Ab and 08-208Bb) that produced the PCR amplicon of 555 pb as well as another amplicon of 556 bp indicating that they harbored both *cdt* III and II types. Isolates 08-244Ba and 08-244Bb (from the same patient) only produced a PCR amplicon of 555 pb belonging to *cdt* type III. No amplification was obtained with primers used to detect the *cdt* type V in any of the clinical *E. coli* isolates. Furthermore, *cdt*^−^ isolate 11-85D did not produce any amplicon using the different set of primers.

These results indicate that the *cdt* type III (> I > IV > II) was the most frequent *cdtB* type among clinical isolates from children in Merida, Mexico.

### Clinical ***cdt***^+^***E. coli*** isolates cause actin cytoskeleton remodeling

**Fig. 3:**
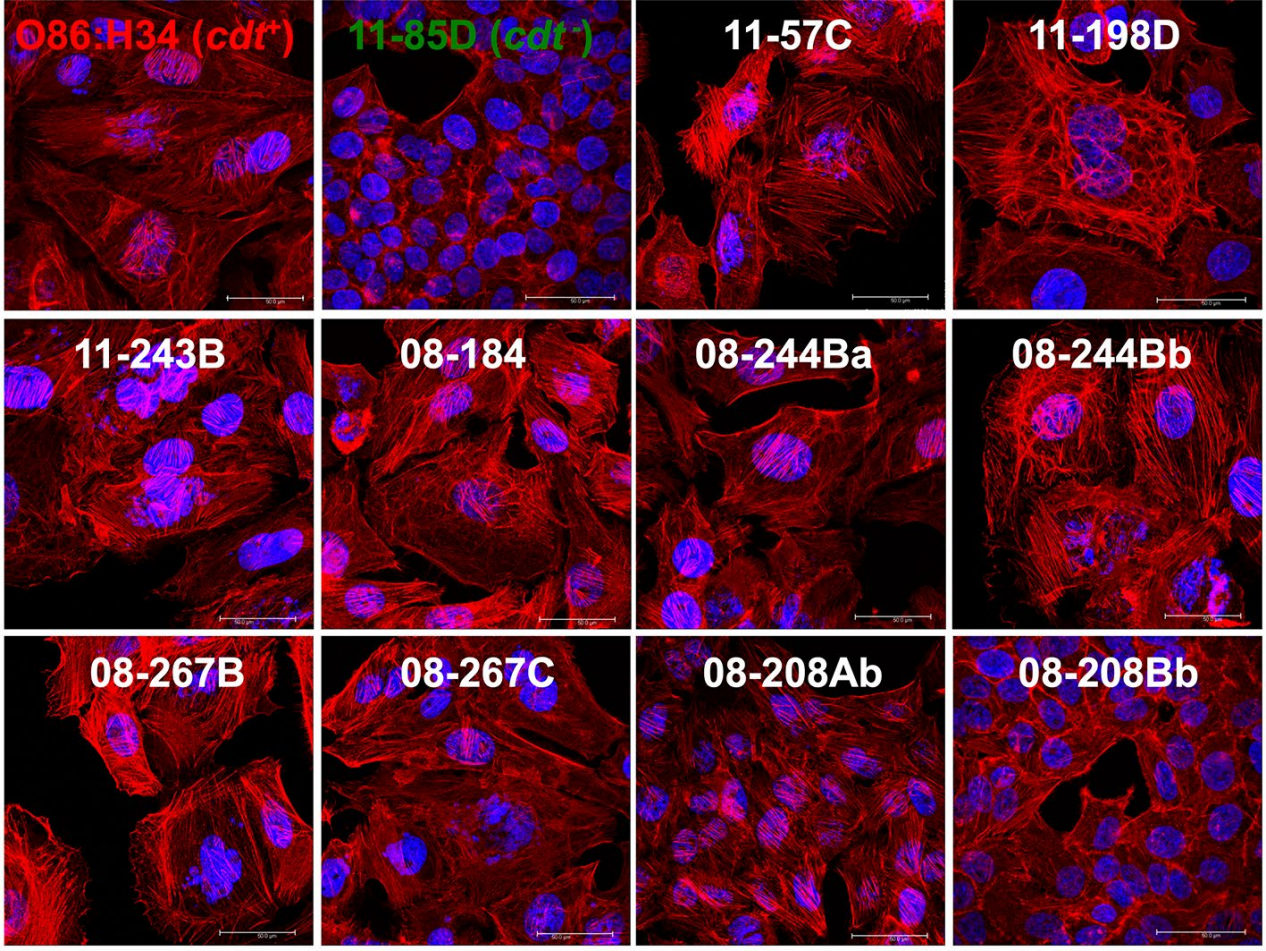
Actin cytoskeletal remodeling induced by the clinical *E. coli* isolates. HEp-2 cells were incubated with filtered bacterial lysates at the appropriated MCC for 24 h. At 48 post-interaction, cells were fixed with 4% PFA. Actin cytoskeleton was revealed by the fluorescent actin staining assay using rhodamine phalloidin (red) and the eukaryotic DNA by TO-PRO-3 staining (blue) and imaged by confocal microscopy. Cells without bacterial lysates (Mock) and with lysates from a *cdt*^−^ strain (11-85D) were used as negative controls, and O86:H34 strain was used as a positive control. Most of the clinical isolates displayed actin cytoskeletal remodeling, including cell and nuclear distention, and strong actin stress fibers. All photographs were taken at a magnification of 950x. Bars, 50 µm

To determine if the *cdt*^+^ *E. coli* isolates cause one of the hallmark effects on host cell cytoskeleton induced by the classical CDT, HEp-2 cells were treated with lysates from the different clinical isolates as well as with lysates from the prototypical O86:H34 strain and a *cdt*^−^ *E. coli* isolate. As expected, untreated cells (mock cells) showed a normal cytoskeleton morphology, with normal stress fibers and cortical actin. Remarkably, all the lysates from the *cdt*^+^ *E. coli* isolates caused cytoskeleton remodeling characterized by strong and long stress fibers displayed by rhodamine phalloidin that detects F-actin, as well as irregular large nuclei detected by TO-PRO-3 staining that detects DNA (Fig. 3). These staining phenotypes also developed in HEp-2 cells treated with the prototypical O86:H34 strain but not with in those with lysates from the *cdt*^−^ isolate. Again, the 08-208Ab isolate showed incipient rearrangement of the actin cytoskeleton, unlike 08-208Bb, which displayed a morphology similar to the mock cells.

### Clinical ***cdt***^+^***E. coli*** isolates cause cell cycle arrest

**Fig. 4:**
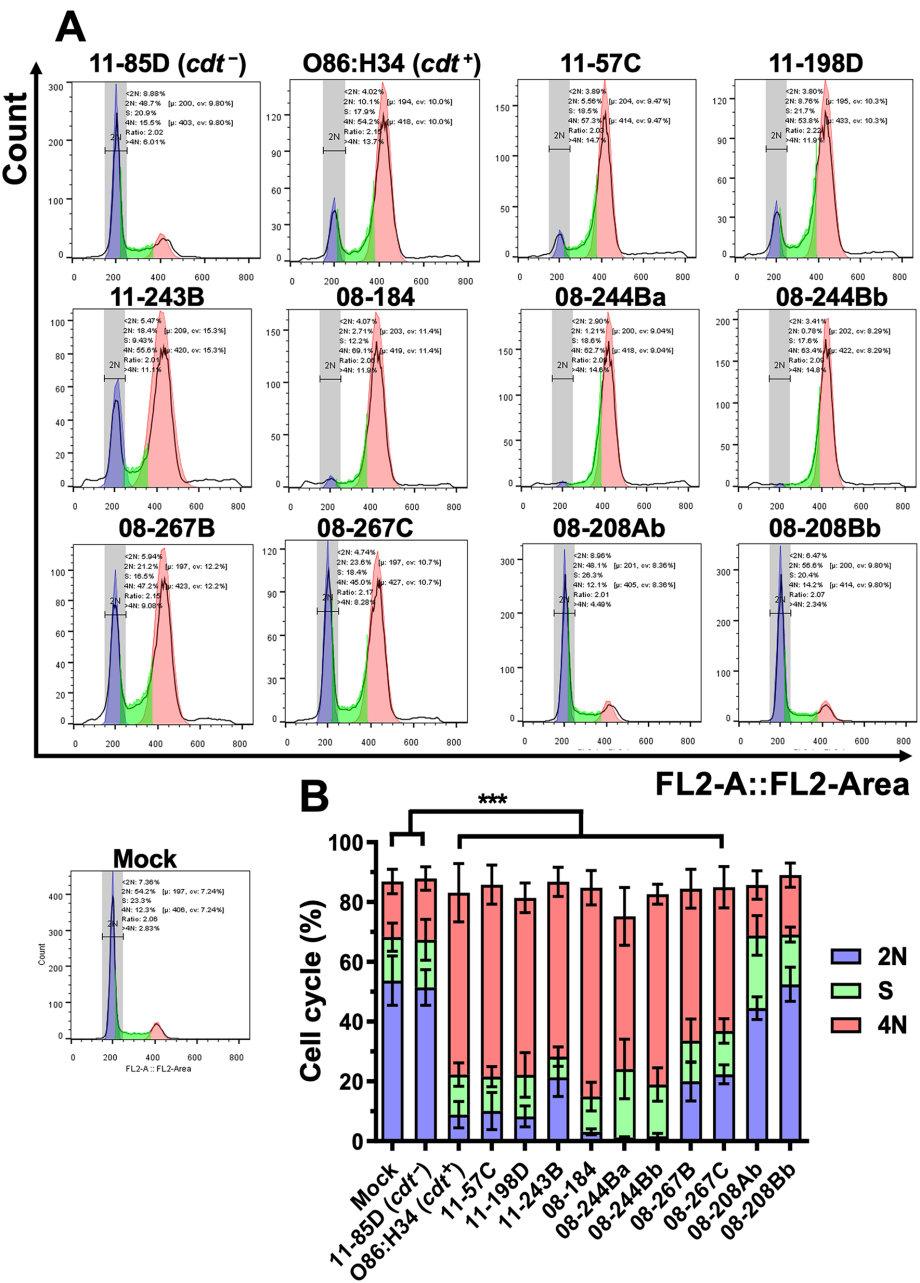
Cell cycle arrest induce by the clinical *E. coli* isolates. HEp-2 cells were incubated with filtered bacterial lysates at the appropriated MCC for 24 h. At 24 h post-interaction, cells were processed for cell cycle analyses by flow cytometry. **A** Cells without bacterial lysates (Mock) and with lysates from a *cdt*^−^ strain (11-85D) were used as negative controls, and O86:H34 strain was used as a positive control. Most of the clinical isolates caused cell cycle arrest (G2/M phase). **B** Cell percentage in the different cell cycle phases (n = 4). p < 0.001. ANOVA One-way Bonferroni’s multiple comparison test (10,000 cells by condition)

To complete the characterization of the cell damage induced by CDT, we tested the ability to cause cell cycle arrest. HEp-2 cells were treated for 24 h with the different bacterial lysates, and then processed for cell cycle analyses by flow cytometry. As expected, mock cells showed that most of them were in, the 2N, G1 phase (~ 54%) and few of them in 4N, G2 (~ 12%), while lysates from the *cdt*^−^ isolate were unable to change these cell cycle values (2N, 49% and 4N 15%) (Fig. 4). By contrast, lysates from *cdt*^+^ *E. coli* isolates caused a cell cycle arrest in G2 phase, with a similar potency. The 08-184, 08-244Ba and 08-244Bb isolates were more efficient (the last two are from the same patient); as most of the treated cells were in 4N, G2 phase (69.1, 62.7, and 63.4%, respectively) and only few of them in 2N (4, 2.9, and 3.4%, respectively). The less efficient lysates were 08-267B and 08-267C (both from the same patient) causing an arrest in G2 phase of 47.2 and 45% (in 4N), respectively, while the populations in 2N were of 5.9 and 4.7%, respectively (Fig. 4). Interestingly, two other lysates (11-57C and 11-198D) showed a cell cycle arrest like those of the prototypical O86:H34 strain (~ 54% in 4N) and with about 4% population in 2N. As expected, the two *cdt*^+^ lysates (08-208Ab and 08-208Bb, from the same patient), which had been unable to cause cell distention, did not cause a cell cycle arrest (4N, 12 and 14% and 2N 48 and 56%).

All these data indicate that these clinical *cdt*^+^ *E. coli* isolates are capable of expressing different *cdt* type (I, II, III and IV), all of which cause cell cycle arrest in G2 and, damage to host cells.

### Deletion of *cdtABC* genes (*cdt* type I or II) abolishes CDT effects on epithelial cells

**Fig. 5:**
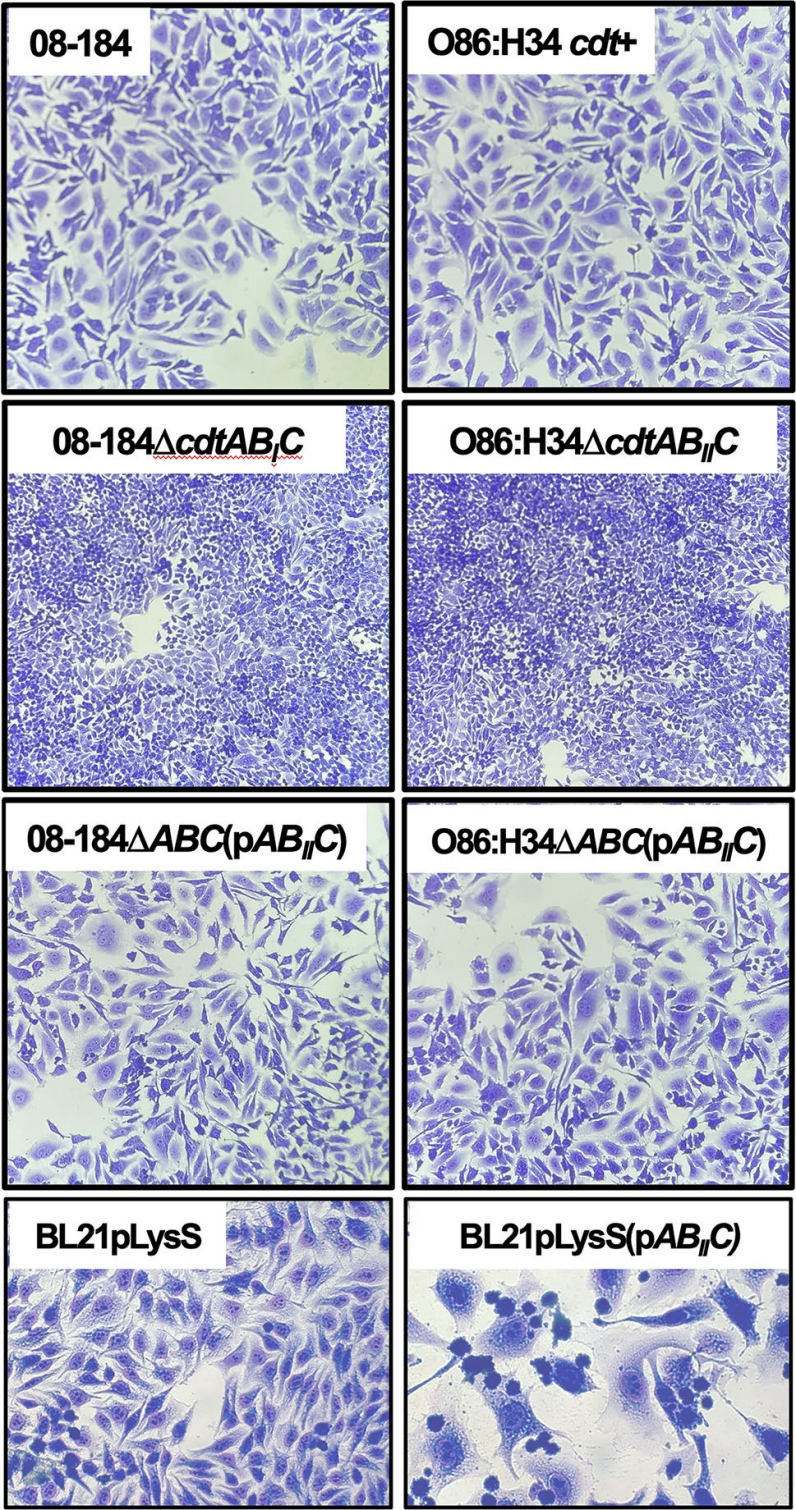
Deletion of *cdtABC* genes in O86:H34 (*cdt* type I) and 08-184 *E. coli* clinical isolate (*cdt* type II) abolish cell distention and their complementation with *cdtAB*_*II*_*C* reestablish the original phenotype. Mutants of *cdtABC* were constructed from the O86:H34 strain and 08-184 *E. coli* clinical isolate to generate O86:H34Δ*cdtAB*_*I*_*C* and 08-184Δ*cdtAB*_*II*_*C*, both were then complemented with a plasmid containing the *cdtAB*_*II*_*C* genes from the 08-184 isolate. Additionally, *cdtAB*_*II*_*C* genes were cloned in a laboratory *E. coli* (BL21[DE3]pLysS). Filtered bacterial lysates from all these constructions and their parentals were incubated with HEp-2 cells at the appropriated MCC for 24 h. After 48 h post-interaction, cells were fixed with methanol and stained with Giemsa, then analyzed by light microscopy

To delete *cdtABC* genes, we first sought for antibiotic resistance of the clinical *cdt*^+^ *E. coli* isolates, mainly in those used to construct mutants. All the *cdt*^+^ *E. coli* isolates were resistant to ampicillin, except for 08-184 and 08-244Ba, which were also susceptible to kanamycin and chloramphenicol (Table 3) as the prototypical O86:H34 strain. In addition to ampicillin, some isolates were resistant to chloramphenicol (11-243B) or kanamycin (11-85D). We thereby, tried deleting *cdtABC* in the prototypical strain and the 08-184 and 08-244Ba isolates by homologous recombination [20]. We successfully constructed two *cdtABC* mutants in the prototypical O86:H34 strain (*cdt* type I) and isolate 08-184 (*cdt* type II). Consistently, both mutants 08-184Δ*cdtAB*_*II*_*C* and O86:H34Δ*cdtAB*_*I*_*C* were unable to cause cell distention and looked like the mock cells (Fig. 5). As expected, the complementation of the mutant 08-184Δ*cdtAB*_*II*_*C* with a plasmid containing the *cdtAB*_*II*_*C* genes (p*AB*_*II*_*C*) reestablished the cell distention effect caused by lysates of this isolate. Remarkably, p*AB*_*II*_*C* from isolate 08-184 (*cdt* type II) was capable complementing the mutant O86:H34Δ*cdtAB*_*I*_*C*, which contained *cdt* type I, to cause a cell distention phenotype similar to its parental *cdtAB*_*I*_*C* genes; *cdtAB*_*II*_*C* has shown here to be more potent than *cdtAB*_*I*_*C* (see Table 3 and Fig. 4). Furthermore, transformation of a laboratory *E. coli* (BL21[DE3]pLysS) strain with the plasmid p*AB*_*II*_*C* caused its lysates to elicit a strong cell distention phenotype (Fig. 5).

**Table 3:** Clinic *E. coli* isolates cause cytotoxic effects at different minimum cytotoxic concentration (MCC)^a^

Patient	Strains/clinical isolates	*cdt* type	MCC (μg/ml)	Antibiotic Sensitivity				
				Kam			Amp	Chl
–	11-85D	*–*	350^c^	R		R		S
–	EPEC O86:H34^b^	I	100	S		S		S
1	11-57C	I	50	S		R		S
2	11-198D	I	150	S		R		S
3	11-243B	I	200	S		R		R
4	08–184	II	50	S		S		S
5	08-244Ba	III	100	S		S		S
	08-244Bb	III	75	S		R		S
6	08-267B	IV	350	S		R		S
	08-267C	IV	300	S		R		S
7	08-208Ab	II, III	350	S		R		S
	08-208Bb	II, III	350	S	R			S

*R* resistant, *S* sensitive

^a^Minimal concentration of bacterial lysates needed to cause cell distention

^b^EPEC O86:H34: CDT-producing enteropathogenic *E. coli* reference strain

^c^Without cytotoxic activity

**Fig. 6:**
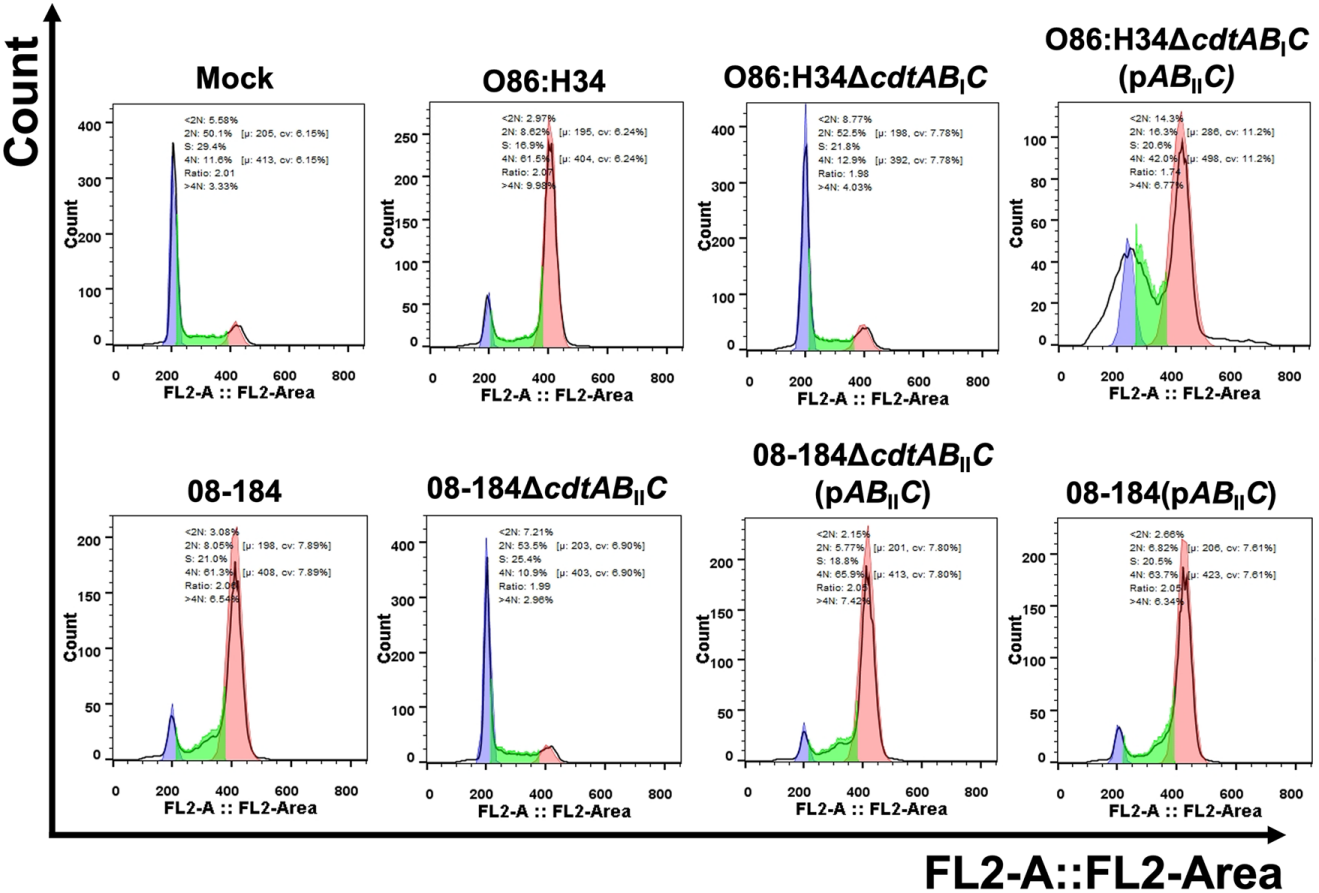
Deletion of *cdtABC* genes in O86:H34 (*cdt* type I) and 08-184 *E. coli* clinical isolate (*cdt* type II) abolish cell cycle arrest and their complementation with *cdtAB*_*II*_*C* (from 08 to 184) reestablish the original phenotype. HEp-2 cells were incubated with filtered bacterial lysates at the appropriated MCC for 24 h. At 24 h post-interaction, cells were processed for cell cycle analyses by flow cytometry. Cells without bacterial lysates (Mock) was used as a negative control and O86:H34 strain was used as a positive control. Cell cycle arrest (G2/M phase) was compared among O86:H34 strain, O86:H34Δ*cdtAB*_*I*_*C* and O86:H34Δ*cdtAB*_*I*_*C*(p*AB*_*II*_*C*) as well as between 08-184 *E. coli* clinical isolate, 08-184Δ*cdtAB*_*II*_*C* and 08-184Δ*cdtAB*_*II*_*C*(p*AB*_*II*_*C*). From 20,000 cells by condition

To corroborate these data, parental bacteria and their mutants were lysed and incubated with epithelial cells and the cell cycle arrest was assessed. As expected, lysates from 08-184Δ*cdtAB*_*II*_*C* and O86:H34Δ*cdtAB*_*I*_*C* were unable to cause cell cycle arrest (4N, 11 and 13%, respectively) and this effect was reestablished when both mutants were complemented with the plasmid p*AB*_*II*_*C* (4N, 66 and 42%, respectively) (Fig. 6).

These data indicate that in at least one of the clinical *cdt*^+^ *E. coli* isolates the *cdtABC* genes are required for causing cytolethal distending effects due to cell cycle arrest similar to the prototypical strain. Furthermore, *cdt* types can be exchanged to cause cell distention and cell cycle arrest. All these strongly suggest that in all clinical isolates *cdtABC* genes are necessary and sufficient to cause the cytolethal distending pathology.

### Few ***cdt***^+^***E. coli*** isolates belong to the EPEC pathotype

**Fig. 7:**
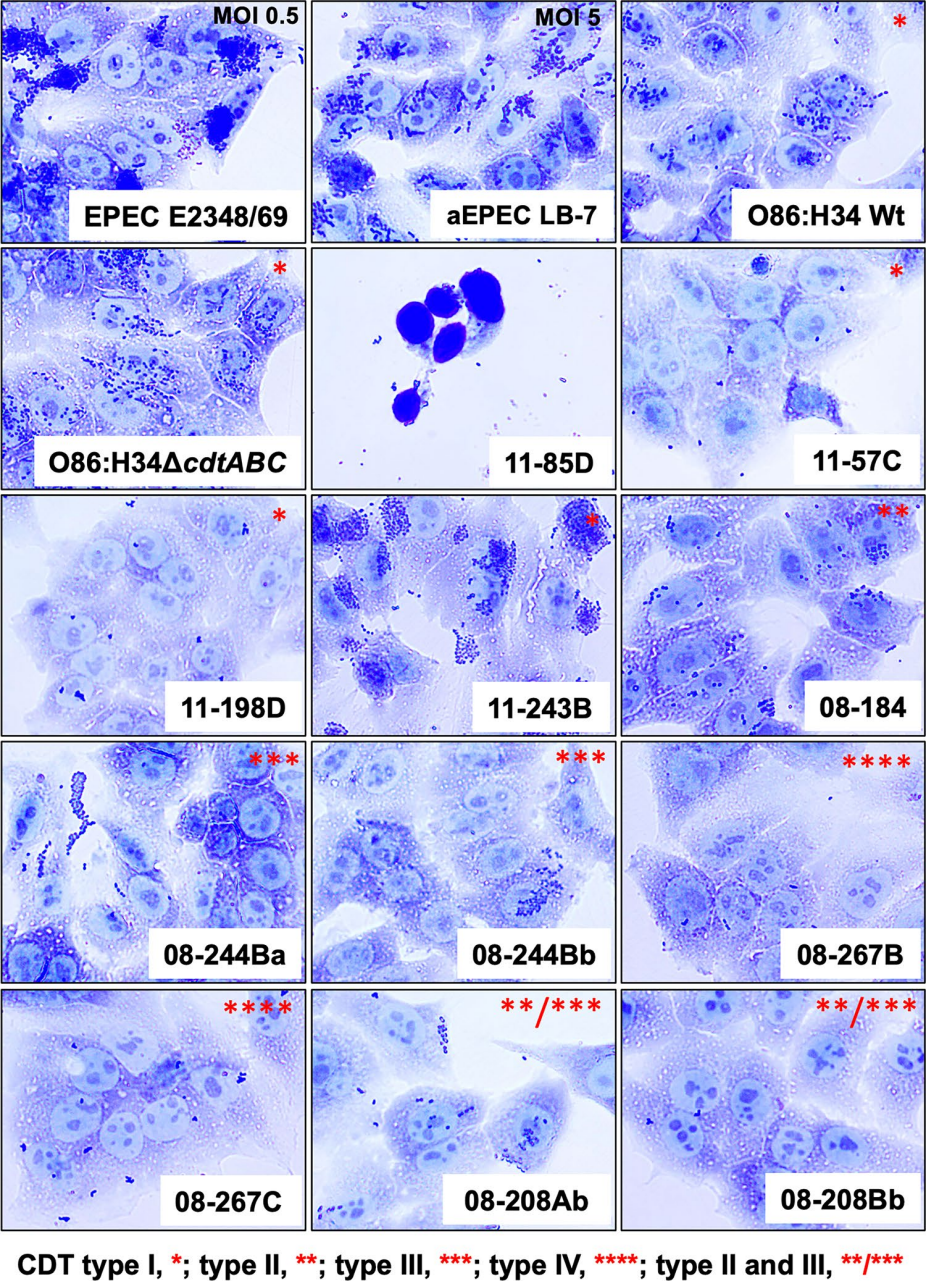
Adherence patterns of the CDT-producing *E. coli* clinical isolates. HEp-2 cell monolayers (70% confluence) were grown on Lab-Tek chamber slides. Bacterial cultures (3.2 × 10^4^ EPEC, 64 × 10^4^ aEPEC, 160 × 10^4^ clinical isolates) was placed into each well, and the plates were incubated for 3 h in a humid, 5% CO_2_ atmosphere. Each *E. coli* clinical isolate (as displayed in each panel) shows also the *cdt* type harbored

Adhesion assays of the clinical *cdt*^+^ *E. coli* isolates on HEp-2 cells were performed to find correlations among cell damage, *cdt* type, and adherence patterns. We used EPEC E2348 (localized HEp-2 cell adhesion: with *bfp*) and the atypical EPEC LB7 (localized-like adhesion: without *bfp*) as well as the prototypical O86:H34 strain, which has been characterized as an EPEC as positive controls. As expected, these three strains displayed a localized or localized-like adhesion consistent with the EPEC pathotype. Among the clinical *cdt*^+^ *E. coli* isolates, 11-243B showed localized adhesion, while 08-184, 08-208Ab (but not 08-208Bb) and both 08-244Ab and 08-244Bb showed localized-like adhesions. The latter was similar to the localized-like adhesion pattern of the O86:H34 strain (Fig. 7), while the other six diarrheagenic isolates did not show a defined adherence pattern. Remarkably, a FAS assay after 6 h of incubation (to promote the formation of aEPEC pedestal) showed that only the 11-243B, 08-184 and 08-208Ab isolates showed actin polymerization at attachment site of bacteria in host cell (pedestal structures) as those displayed by the reference EPEC strains, EPEC E2348, EPEC LB7 and O86:H34 (Suppl. Figure 1). These data indicate that 08-244Ba and 08-244Bb isolates did not form pedestal even when both displayed a localized-like adherence pattern suggesting a deletion in some important gene on the pathogenicity island LEE. Interestingly, 11-243B isolate displayed a better adherence pattern (localized) and harbored *cdt* type I (Fig. 7) but did not have a greater capacity to induce cell distention (see Fig. 1) or cell cycle arrest (see Fig. 4). 11-198D isolate, which showed the lowest bacterial adhesion patterns and harbored *cdt* type I, were the most potent in causing cell cycle arrest. In fact, the cell cycle arrest induced by these lysates was similar to that induced by the prototypical O86:H34 strain, which harbored *cdt* type I and displayed a localized-like adherence pattern (Fig. 7). Interestingly, isolate 08-184, which displayed a localized-like adhesion pattern and harbored a *cdt* type II, was more potent in causing cell cycle arrest than the prototypical strain O86:H34, despite requiring less lysates to cause the full effects (50 versus 100 µg/ml). Interestingly, 11-85D, a *cdt*^−^ isolate that did not cause CDT effects, displayed a strong cytophatic effects in the adherence assays, suggesting that it might harbor other virulence factors (Fig. 7).

These data suggest that there is no relationship among cell damage, *cdt* type, and adherence pattern in the clinical *cdt*^+^ clinical isolates and that, at least in this small number of samples, *cdt* type II caused CDT pathological phenotypes more efficiently than other *cdt* types.

## Discussion

*Escherichia coli* positive for *cdt* genes by PCR (*cdt*^+^) have been identified in both diarrheagenic and uropathogenic *E. coli* strains, as well as in those that do not belong to any recognized pathotype [13, 17, 18]. However, few studies have addressed the cell damage induced by these diverse *cdt*^+^ *E. coli* isolates or the effects of the CDT toxin on epithelial cells in these various *E. coli* genetic backgrounds. Here, we show that the 10 *cdt*^+^ *E. coli* isolates obtained as the sole pathogen from seven children with acute diarrhea produce different *cdt* types (I to IV). These different *cdt* types cause similar cell distention, actin cytoskeleton remodeling, increased nuclei size, and cell cycle arrest in G2 phase, although the minimum concentration to cause these effects differed. Further characterization of one of the *cdt*^+^ isolates containing *cdt* type II (*cdtAB*_*II*_*C*), which exhibited the most potent *cdt* type, showed that *cdt* genes are necessary and sufficient to cause cytolethal distending pathology. The absence of these effects in *cdtAB*_*I*_*C* (in the prototypical O86:H34 strain) and *cdtAB*_*II*_*C* (in the 08-184 isolate) mutants was complemented in both by plasmid *cdtAB*_*II*_*C* genes to cause cell distention, actin cytoskeleton remodeling, and cell cycle arrest. Furthermore, *cdtAB*_*II*_*C* genes were able to transform a laboratory *E. coli* strain to cause cytolethal distending pathology. Interestingly, we did not find a relationship among cell damage, *cdt* type, and adherence patterns in the *cdt*^+^ isolates. Thus, these data indicate that *cdt*^+^ *E. coli* isolates have the potential to cause serious epithelial cell damage leading to cell death and clinical diarrhea.

CDTs are encoded by three linked genes (*cdtA*, *cdtB* and *cdtC*), and CdtB is the toxin subunit with DNase I-like activity. The three genes must be present in the bacterium to produce an active cytotoxin [21]. Here we have shown that all the *cdt*^+^ *E. coli* isolates from children with acute diarrhea produced the main CDT effects, except for two isolates that came from the same patient. This indicates that all isolates produce the three functional proteins needed to assemble the heterotrimeric toxin responsible for cell distention and cytoskeleton remodeling, including enlargement of nuclei (Figs. 1 and 3). The PCR primers typically used to identify *cdt*^+^ *E. coli* isolates hybridize with the *cdtB* gene [4]. However, all three functional products of *cdtA*, *cdtB* and *cdtC* genes are required to cause the cytotoxic effects [21]. Our findings are relevant since other CDT-producing bacteria lack a complete set of *cdtABC* genes, and are heterogeneous in their *cdt* genes and virulence, as found in about 48% of the Iranian *Campylobacter* population (33 *Campylobacter* spp. isolated from patients with diarrhea) [22]. In another study, *Campylobacter* isolates from three distinct origins (wild birds, broiler chickens, and humans) showed that the sequence of *cdtABC* is highly conserved among broiler and human isolates. However, a high diversity of *cdtABC* alleles was found among wild bird isolates, including several alleles that do not produce functional CDT [23]. Importantly, all our *cdt*^+^ *E. coli* isolates showing full CDT activity on epithelial cells, were obtained from children who presented watery diarrhea with high stool output (range 7–20 stools/24 h); five also had fever of 38 °C or more and four presented vomiting. Dehydration was present in four patients, one of whom had hypovolemic shock; one child also presented hyponatremia and hypokalemia [19]. A recent study 422 *C. jejuni* and 84 *C. coli* genome sequences reported that key virulence genes, such as *cdtABC* (*C. jejuni*) and *cadF* (encoding a binding protein) were prevalent (> 90% presence) but did not correlate with disease severity or hospitalization [24].

 Remarkably, *cdt*^+^ *E. coli* isolates harbored different types of *cdt*, mainly type I (3 isolates), II (1 isolate), III (2 isolates), and IV (2 isolates) and both II and III (2 isolates) (Fig. 2). Further characterization exposed two interesting findings: there is no relationship between CDT-induced cell damage, bacterial adhesion and the type of CDT involved, and there was no major difference between the type of *cdt* and the induced epithelial phenotype by CDT (cell distension, actin cytoskeleton remodeling, nuclear size, and cell cycle arrest). Although *cdt* type II causes a strong epithelial phenotype, it is not possible to reach a conclusion as only one isolate harbored this type of *cdt*. On the other hand, a study of CDT-producing *E. coli* from Japanese children with diarrhea, showed that of the 35 samples that were positive for the *cdtB* gene, 21 were positive for *cdt*-I, three for *cdt-*II, four for *cdt*-III, three for *cdt*-IV, and four for *cdt*-V; all strain produced an active CDT that caused cell distention in HeLa cells. However, the authors argue that most of these isolates do not belong to a conventional enteropathogenic pathovar and thus, differ from strains isolated in developing countries [13]. We found only two enteropathogenic pathotypes among our ten isolates (both produced the CDT effects and were FAS positive). Adherence patterns of EPEC were seeking because these *cdt*^+^ isolated were selected from a previous study where only *bfp* and *eaeA* genes were detected but no other genes associated with other diarrheagenic pathotypes [19]. One was a typical EPEC and another an atypical EPEC, but interestingly, the CDT effects of these two isolates were not highly different from those of *E. coli* isolates with undefined pathotypes. Furthermore, despite their strong bacterial adhesion patterns (localized and localized-like), the cytolethal distending pathology was similar to those isolates with almost no adhesion (Fig. 7). In contrast, a study regarding cytotoxic activity found that the CDT-producing atypical EPEC strains appeared to be less toxic than the CDT-producing typical EPEC strains; interestingly, only *cdt* type I, IV and V were found in 10 *cdtB* gene-positive strains [25]. In our study, isolate 08-184 harboring *cdt* type II with a localized-like adherence pattern belonging to an atypical EPEC showed stronger CDT activity. These data strongly suggest that CDT delivery to epithelial cells by *E. coli* is not mediated by bacterial adhesion. Another interesting finding was that only one patient carrying *cdt*^+^ isolates (08-208Ab and 08-208Bb) was negative for the cytolethal distending pathology, despite both harboring two *cdt* types, II and III, simultaneously. A possible explanation is that production of two different types of *cdt* could interfere with the assembling of a functional heterotrimeric toxin. This is not consistent, however, with a study of 75 *cdtB*^+^ *E. coli* isolates, in which *cdt*-II and -III were detected simultaneously in 30.8% all of which exhibited a characteristic of CDT cytopathic effect in the CHO cell assay [11]. Thus, more studies are required to explore the relationship between the *cdt* type and morphophysiological effects on epithelial cells. It is possible that individual members of the CDT superfamily could interact with host cells by distinct mechanisms [26] or by differences in their subunits [27].

The effects of *cdt*^+^ isolates on the cell cycle are consistent with our results for cell distention, actin cytoskeleton remodeling, and nuclei enlargement. Thereby, all the isolates causing classical CDT morphological effects also caused cell cycle arrest. Interestingly, one isolate (08-184, *cdt*-II) showed a higher potency of cell cycle arrest than the other isolates, including the prototypical O86:H34 strain. This bacterial isolate was used to demonstrate that a *cdtAB*_*II*_*C* mutant is unable to cause this cytolethal distending pathology and that a transformed laboratory *E. coli* strain with these genes successfully reproduced the classical CDT effects, cell distention (Fig. 4) and cell cycle arrest (Fig. 5). Several studies have shown that the distention of CDT-intoxicated cells is associated with the promotion of actin stress fibers in the cytoskeleton, which results from the activation of DNA-damage checkpoints [28, 29]. These data together with the complementation of the *cdtABC* mutant in the prototypical O86:H34 strain (*cdt*-I) with the plasmid containing *cdtABC* (*cdt*-II) shed light on the structure–function relationship of these toxins in *E. coli*. This also strongly suggests a high capacity for assembling this toxin in the *E. coli* genetic background.

In conclusion, children with acute diarrhea harboring *cdt* + *E. coli* isolates as the sole pathogen not only showed strong clinical signs and symptoms of sufficient severity to warrant hospitalization [19]. But these isolates also showed the hallmark CDT effects on epithelial cells, which included cell distention, strong F-actin cytoskeleton stress fibers (including nuclei enlargement), and cell cycle arrest (4N cells), in G2/M phase. Furthermore, myriad *cdt* types were isolated from these seven children (10 isolates), except for *cdt*-V. Interestingly, two isolates from the same patient harbored two simultaneous *cdt* types, II and III but did not exhibit hallmark CDT effects. Remarkably, one isolate (08-184) demonstrated the highest potency to cause the CDT effects (*cdt*-II and localized-like adherence pattern), mainly in cell cycle arrest, but not the other EPECs (one of strong localized adherence, and other of localized-like adherence). These data challenge the belief that bacterial adhesion may be an important factor in this toxin delivery. Expression of these mutated genes (*cdtAB*_*II*_*C*) in this isolate was clearly demonstrated by complementation experiments using a plasmid containing these genes. These genes (*cdt* type II) also complemented the CDT effects in a mutant of the prototypical strain (originally harboring a *cdt* type I) and were also able to transform a laboratory *E. coli* strain to display the CDT intoxicated phenotype. This work also supports the previous proposals that CDT-producing *E. coli* (CTEC) could represent a new category of diarrheagenic *E. coli*. To our knowledge, this is the first study to assess the association of CDT-producing bacteria isolated from patients with diarrheal patients and CDT activities on epithelial cells in Mexico.

## Supplementary Information

Below is the link to the electronic supplementary material.

Supplementary file1 (TIF 8332 KB) Suppl. Fig. 1. F-actin staining assays to detect actin-rich pedestals formed by EPEC bacteria. HEp-2 cell monolayers (70% confluence) were grown on Lab-Tek chamber slides. Bacterial cultures (3.2×10^4^ EPEC, 64×10^4^ aEPEC, 160×10^4^, 320×10^4^ clinical isolates; for high and low adherence) were placed into each well, and the plates were incubated for 6 h in a humid, 5% CO2 atmosphere. Nuclei and bacterial DNA were stained using DAPI (blue) and the F-actin cytoskeleton was stained using rhodamine-phalloidin (red). Each *E. coli* clinical isolate (as displayed in each panel) shows also the *cdt* type harbored. Arrows point out the actin pedestals.

## Data Availability

No datasets were generated or analysed during the current study.
