# Supplementary figures and images for: Cytolethal distending toxin-producing Escherichia coli clinical isolates from Mexican children harbor different cdt types causing CDT-induced epithelial pathological phenotypes
Source: Med Microbiol Immunol. 2025 Feb 2;214(1):7. doi: 10.1007/s00430-025-00816-4 (PMC11788229; doi:10.1007/s00430-025-00816-4)

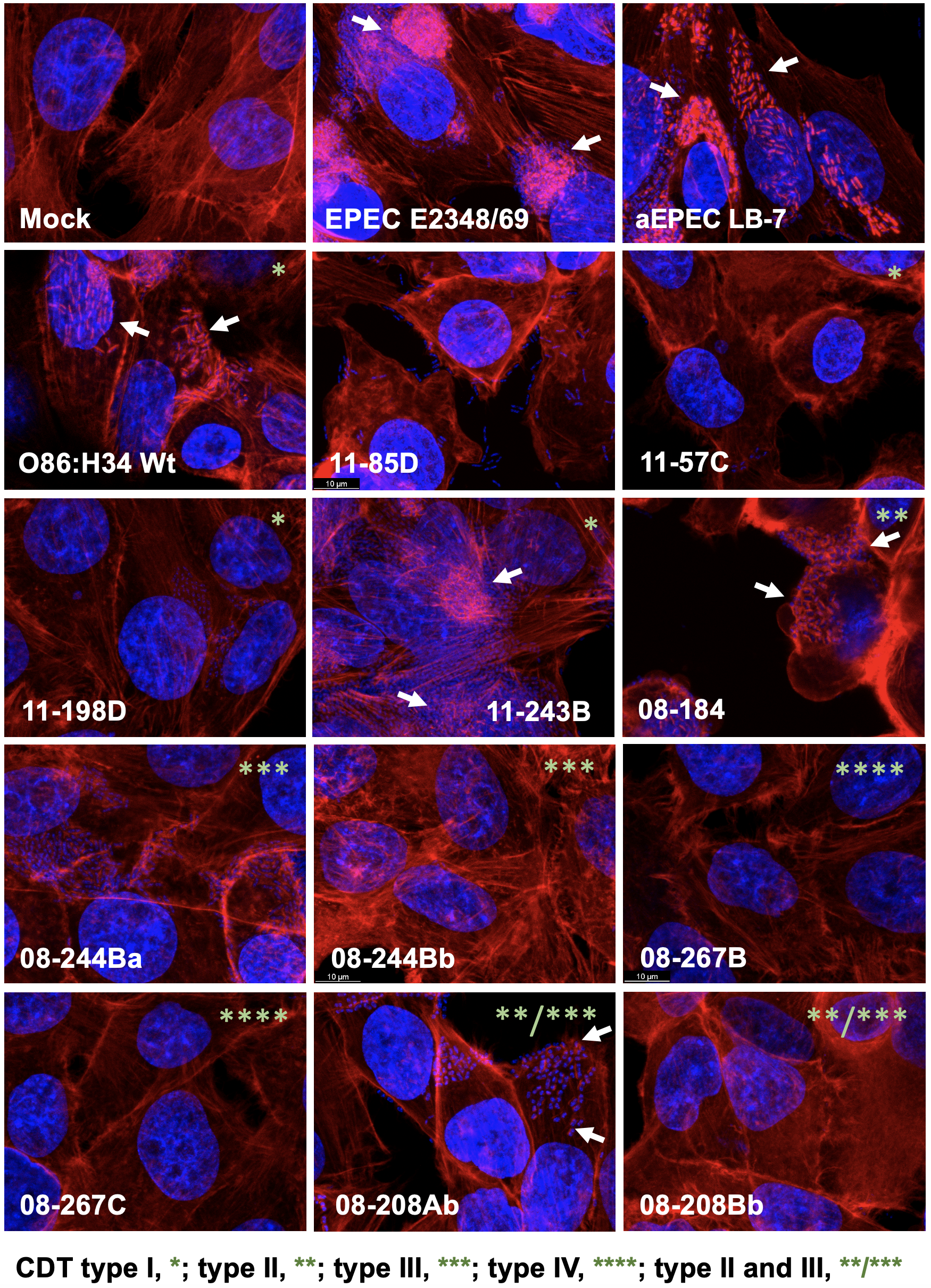

Supplement: Supplementary file 1 — Supplementary file1 (TIF 8332 KB) Suppl. Fig. 1. F-actin staining assays to detect actin-rich pedestals formed by EPEC bacteria. HEp-2 cell monolayers (70% confluence) were grown on Lab-Tek chamber slides. Bacterial cultures (3.2×10^4^ EPEC, 64×104 aEPEC, 160×104, 320×104 clinical isolates; for high and low adherence) were placed into each well, and the plates were incubated for 6 h in a humid, 5% CO2 atmosphere. Nuclei and bacterial DNA were stained using DAPI (blue) and the F-actin cytoskeleton was stained using rhodamine-phalloidin (red). Each E. coli clinical isolate (as displayed in each panel) shows also the cdt type harbored. Arrows point out the actin pedestals. [file 430_2025_816_MOESM1_ESM.tif]
